# Preoperative pain catastrophizing and postoperative pain after total knee arthroplasty: a prospective cohort study with one year follow-up

**DOI:** 10.1186/s12891-016-1073-0

**Published:** 2016-05-17

**Authors:** Lise Husby Høvik, Siri Bjørgen Winther, Olav A. Foss, Kari Hanne Gjeilo

**Affiliations:** Clinic of Anaesthesia and Intensive Care Medicine, St. Olavs Hospital, Trondheim University Hospital, Postboks 3250, N-7006 Trondheim, Norway; Orthopaedic Research Center, Orthopaedic Department, St. Olavs Hospital, Trondheim University Hospital, Trondheim, Norway; Department of Neuroscience, Faculty of Medicine, Norwegian University of Science and Technology, Trondheim, Norway; Department of Cardiothoracic Surgery, St. Olavs Hospital, Trondheim University Hospital, Trondheim, Norway; Department of Cardiology, St. Olavs Hospital, Trondheim University Hospital, Trondheim, Norway; National Competence Centre for Complex Symptom Disorders, St. Olavs Hospital, Trondheim University Hospital, Trondheim, Norway; Department of Circulation and Medical Imaging, Faculty of Medicine, Norwegian University of Science and Technology, Trondheim, Norway

**Keywords:** Total knee arthroplasty, Pain catastrophizing, Pain, Fast-track surgery

## Abstract

**Background:**

Pain relief is likely to be the most important long-term outcome for patients undergoing total knee arthroplasty (TKA). However, research indicates that persistent pain (> 3 months) is a considerable problem, affecting up to 34 % of patients. Pain catastrophizing might contribute to acute and persistent pain experienced after surgery. The primary aim of the present study was to examine the association between preoperative pain catastrophizing and postoperative pain in patients undergoing TKA up to one year after surgery. Second, we wanted to investigate a possible shift in postoperative catastrophizing.

**Methods:**

In this prospective cohort study, 71 TKA patients were included consecutively between January and June 2013. Pain was assessed with the Brief Pain Inventory (BPI) and the item “average pain” was used as the main outcome. Pain catastrophizing was measured by the Pain Catastrophizing Scale (PCS). Questionnaires were completed prior to surgery (baseline) and at two days, two weeks, eight weeks and one year postoperatively.

**Results:**

Mean (SD) preoperative pain score was 5.4 (2.2), reduced to 2.9 (2.3) after eight weeks and 2.4 (2.4) after one year (*p* < 0.001). The overall median preoperative PCS score was 17.0 (7.8–28.3). The overall model estimated PCS mean score was 7.6 at eight weeks and 6.5 at one year follow-up. The results at eight weeks and one year follow-up were both significantly lower than the preoperative value (*p* < 0.001). The preoperative PCS score was not associated with the postoperative pain score (*p* = 0.942), while preoperative pain was a significant covariate in the mixed linear model (*p* < 0.001).

**Conclusions:**

No associations were found between preoperative pain catastrophizing and pain eight weeks or one year after surgery. The decrease in PCS-scores challenges evidence regarding the stability of pain catastrophizing. However, larger studies of psychological risk factors for pain after TKA are warranted.

## Background

Severe knee osteoarthritis is the most common reason for patients to seek knee replacement. Total knee arthroplasty (TKA) improves function and reduces pain for the majority of patients, and pain relief is likely to be the most important long-term outcome [1]. However, research indicates that persistent pain is a considerable problem, affecting up to 34 % of patients [2–6]. Surgery is a known risk factor for developing chronic pain, most often defined as pain present for at least three months [7]. Studies have found that almost 20 % of patients are not satisfied one year after surgery [2, 4, 8]. The main reason appears to be persistent pain during activities of daily living [5, 9–11].

TKA is considered a painful procedure, and pain after hospital discharge is an ongoing challenge despite multimodal approaches to pain management [12]. Pain after surgery seems to be the main factor limiting early mobilization [13]. Successful rehabilitation after TKA depends on patients’ recovery efforts and ability to cope with pain. Physical activities targeted towards regaining muscle strength are important to reduce the risk of postoperative complications such as prolonged stiffness, persistent pain and diminished function [14–16].

Recent studies evaluating the predictors of persistent pain after TKA have suggested that some psychological variables might predispose individuals to a negative pain-related outcome after surgery [10, 17]. Anxiety and depression have been frequently evaluated, and the role of pain catastrophizing is increasingly being considered [18, 19]. Pain catastrophizing is characterized by the tendency to magnify the threat from pain stimulus, to feel helpless in the context of pain and to ruminate about the pain experience [20]. Catastrophizing in a pain context can reduce the patient’s adherence to the training program and appears to have a negative impact on pain severity after TKA [21]. Preoperative pain catastrophizing has been a strong predictor of postoperative pain in several studies of knee replacement surgery [21–24]. However, other studies find weak or no association [25, 26] and a recent systematic review found that only a few studies have followed patients beyond three months [18].

The primary aim of this study was to explore the association between preoperative pain catastrophizing and postoperative pain up to one year after surgery in patients undergoing primary TKA. Second, we wanted to investigate a possible shift in postoperative pain catastrophizing.

## Methods

This prospective cohort study was conducted at the Department of Orthopaedic Surgery at St. Olavs University Hospital in Trondheim, Norway between January and June 2013. Baseline questionnaires of pain and pain catastrophizing were given to the patients for self-administration at the pre-surgical evaluation, within two weeks prior to surgery and the follow-up assessment of pain two days after surgery, when hospitalized. Further follow-up was performed at two weeks, eight weeks and one year after surgery. The participants were sent one postal reminder after two weeks if the questionnaires were not returned. Those who did not respond were considered non-responders.

The study was approved by the Regional Committee for Medical and Health Research Ethics in Central Norway (no. 2012/1698/REK midt). The patients received oral and written information about the study, and written informed consent was obtained from each patient before inclusion.

### Patient population

The patients followed a standardized fast-track procedure for hip and knee arthroplasty implemented in our department [27]. All patients scheduled for primary TKA were consecutively included after approving participation. The first author was responsible for patient inclusion and administration of the follow-up questionnaires. Criteria for exclusion were: lack of ability to write or read Norwegian, cognitive impairments (inability to provide informed consent) or refusal to participate.

### Measures

Pain was measured by the Norwegian version of the Brief Pain Inventory (BPI) Short form [28]. The BPI is a short, self-administered questionnaire designed to assess the intensity of pain and the impact of pain on daily function during the past 24 h [29–32]. The BPI consists of eleven items, of which four questions are related to pain intensity; pain now, worst, least and average pain. The patients are asked to rate their pain on a numerical rating scale (NRS), where 0 = no pain and 10 = worst imaginable pain. The primary outcome was the item “average pain” measured at two days, two weeks, eight weeks and one year follow-up.

Pain catastrophizing was measured by the Norwegian version of the Pain Catastrophizing Scale (PCS) [33, 34]. The PCS consists of thirteen items describing the thoughts and feelings that patients may experience when they are in pain [20]. Patients rate their thoughts regarding pain using a five-point Likert scale with the endpoints 0 (“not at all”) and 4 (“all the time”). It is self-administered and the PCS total score is calculated by summing the thirteen- item responses ranging from 0 (no catastrophizing) to 52 (severe catastrophizing). PCS is considered to have high internal consistency with Chronbach’s α reported to be 0.87 [34]. Individuals who score above 30 on the PCS are considered to be at high risk for developing chronicity and considered suitable candidates for risk-factor targeted intervention programs [20]. Pain catastrophizing was measured at baseline, at eight weeks- and one year follow-up.

### Statistical analysis

Statistical calculations were performed using IBM SPSS Statistics version 21. Visual inspections of Q-Q plots were used to determine whether or not the data were normally distributed.

The preoperative PCS scores were not normally distributed and are therefore presented as medians (with quartiles). Preoperative pain scores were normally distributed and are presented as means (SD). The Mann–Whitney *U*-test was used when comparing preoperative PCS scores and independent samples Student’s *t*-test when comparing preoperative pain between the genders. Average pain and PCS scores at the follow-up assessments were analyzed using mixed linear models to account for dependency caused by repeated measures data. All baseline characteristics presented in Table 1 were initially considered as potential covariates. Preoperative pain score and gender were used as covariates in the model based on the result from directed acyclic graphs analyses [35]. The residuals in the model were found to be normally distributed. Bonferroni adjustment was used when comparing differences in average pain between the different follow-up assessments. The level of statistical significance was set at *p* < 0.050.

**Table 1:** Baseline characteristics of the study population (*n* = 71)

Characteristics		Value
Age, mean (SD)		64.8 (10.3)
Female sex, *n* (%)		36 (50.7)
ASA group, *n* (%)	1	8 (11.3)
	2	49 (69.0)
	3	13 (18.3)
	4	1 (1.4)
BMI kg/m^2^, mean (SD)		30.4 (5.4)
Living with others, *n* (%)		49 (69.0)
PCS total, mean (SD)		18.2 (12.1)
BPI average pain, (NRS 0–10), mean (SD)		5.4 (2.2)

*ASA* American Society of Anesthesiologists classification, *BMI* Body Mass Index, *BPI* Brief Pain Inventory, *PCS* Pain Catastrophizing Scale total (0–52), *NRS* Numerical Rating Scale

## Results

**Fig. 1:**
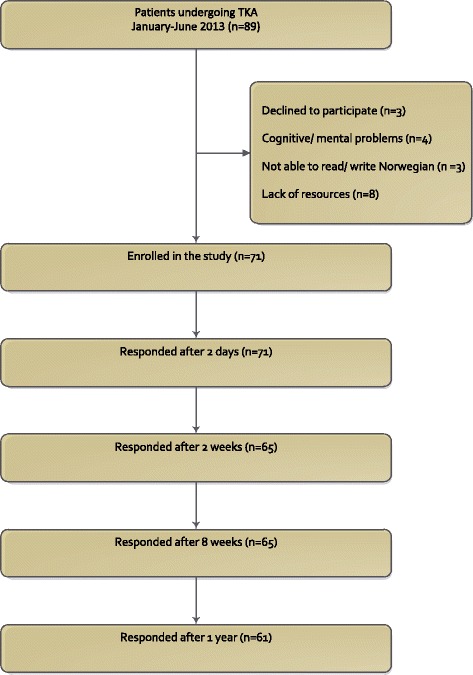
Flow of patients through the study. TKA: Total Knee Arthroplasty

Seventy-one of 89 patients undergoing TKA during the period were included in the present study (80 %). Fig. 1 shows the flow of patients through the study. Baseline and demographic data are presented in Table 1.

The overall median preoperative PCS score was 17.0 (7.8–28.3). The median for females was 17.5 (8.0–27.8) and that for males 17.0 (6.8–32.0). The difference between the sexes was not statistically significant (*p* =0.865). The overall model estimated PCS mean score was 7.6 at eight weeks and 6.5 at one year follow-up. There was no statistical significance between the results at eight weeks and one year follow-up (*p* = 0.290); however both the results at eight weeks and one year follow-up were significantly lower than the preoperative value (*p* < 0.001).

The mean preoperative average pain score was 5.4 (2.2). The females mean score was 6.1 (2.0) and males 4.7 (2.2) with a statistically significant difference (*p* = 0.006). There were statistically significant differences in the pain scores across the follow-up assessments (*p* < 0.001). Differences between results of the follow-up assessments are presented in Table 2.

**Table 2:** Pain score time-trends in 1-year after TKA

	Mean (SD)		Mean difference (95 % CI)	*P*-value
2 days	4.3 (2.0)	2 weeks	0.6 (0.0,1.3)	0.046
		8 weeks	1.4 (0.8,2.2)	< 0.001
		52 weeks	1.9 (1.1,2.7)	< 0.001
2 weeks	3.7 (2.2)	2 days	−0.6 (−1.3,–0.0)	0.046
		8 weeks	0.8 (0.2,1.5)	0.007
		52 weeks	1.3 (0.5,2.1)	< 0.001
8 weeks	2.9 (2.3)	2 days	–1.4 (–2.2,–0.8)	< 0.001
		2 weeks	–0.8 (–1.5,–0.2)	0.007
		52 weeks	0.5 (–0.3,1.2)	0.592
52 weeks	2.4 (2.4)	2 days	–1.9 (–2.7,–1.1)	< 0.001
		2 weeks	–1.3 (–2.1,–1.1)	< 0.001
		8 weeks	–0.5 (–1.2,0.3)	0.592

Pain measured by the item “average pain” in the Brief Pain Inventory (BPI)

TKA (Total Knee Arthroplasty)

Mixed Linear Models to account for dependency caused by repeated measures data

The model adjusted, preoperative PCS score had no statistically significant effect on the postoperative pain score (*p* = 0.942). Females reported a mean postoperative pain score of 3.5 and males reported 3.1 (*p* = 0.323).

Preoperative pain was a significant covariate in the mixed linear model (*p* < 0.001). An increase of 1 in preoperative pain score resulted in an increase of 0.4 in postoperative pain score.

## Discussion

The primary aim of this study was to examine whether the levels of preoperative pain catastrophizing were associated with pain up to one year after TKA. We found no support for such an association. The preoperative catastrophizing had no significant influence on postoperative pain at any of the follow-up assessments.

The PCS is thought to be useful for identifying individuals predisposed to a heightened distress response to painful procedures such as surgery [34]. Recognizing risk factors for acute or persistent pain after knee replacement could allow targeted perioperative treatment, including tailored postoperative pain treatment or individualized postoperative follow-up. Several studies report catastrophizing to be highly correlated with pain related outcomes [17] and pain catastrophizing is frequently mentioned as a contributor to acute and persistent pain after TKA [21–23, 36]. Our results do not support the PCS questionnaire as a tool to identify patients at risk for developing persistent pain after TKA.

Wether the PCS has a predictive value for postoperative pain seems inconclusive [18]. Other studies of TKA [24, 37, 38], as well as other types of surgery [39, 40], are in accordance with our results and have found little or no evidence for an association between pain catastrophizing and postsurgical pain. A prospective cohort study by Banka et al. (2015) even found that high catastrophizing gave decreased odds of postoperative opioid use six weeks after TKA, and found no evidence of an association between preoperative catastrophizing and postoperative pain [25].

Our secondary aim was to investigate a possible shift in pain catastrophizing after surgery. Interestingly, we found a significant reduction in catastrophizing during the follow-up period. The levels of preoperative catastrophizing were relatively high in the present study. Comparable studies of TKA report a wide variety of preoperative PCS scores with mean values from 7.1 to 19.4 [11, 21, 41–43]. High scores of preoperative catastrophizing in our study may be explained by imminent surgery, considering that catastrophizing is related to the tendency to exaggerate the possibility of a catastrophic result [44] as well as the level of preoperative pain. Knee joint arthritis is defined as chronic pain [45] and psychologically robust individuals may have the tendency to catastrophize when experiencing pain in certain situations [41, 44]. Joint replacement is considered a highly effective treatment for reducing pain in patients diagnosed with osteoarthritis [1]. When pain intensity declined, so did the levels of catastrophizing. However, a recent systematic review of patients undergoing TKA, found that pain catastrophizing levels remain stable over time [4]. This could have been caused by differences in research methods, but many studies lack a thorough description of the organization of the patient pathway (patient education, length of stay, physiotherapy follow-up etc.) which may have influenced pain catastrophizing scores.

Reducing pain catastrophizing is highlighted as a key factor in determining successful rehabilitation for pain-related conditions [46] and reduction of catastrophizing is associated with clinical improvement of pain [47]. Catastrophizing shows some degree of stability over time in the absence of interventions but can be context dependent and determined by situational factors [48]. Several intervention studies have examined the possibility of modifying catastrophizing. Psychological and psychosocial interventions [49], education and instruction in self-management skills [50] or exercise and physical therapy [51, 52] were shown to be effective in reducing pain catastrophizing to some degree.

The consequence of pain-related psychosocial risk factors such as pain catastrophizing, seems to be reduced activity or participation in daily activities. Providing assurance that the pain condition does not contain a serious health risk has shown to have an effect on physical activity [53]. Information and education allow patients to re-evaluate the threat they associate with their condition [54]. In our study there were no interventions aimed at reducing catastrophizing. However, all patients in the fast-track program are given the best evidence-based treatment available, targeting patient education, pain relief and early mobilization, which reduces the need for hospitalization [55]. This includes a preoperative educational class and individual information (oral and written) on specific clinical procedures. Our fast-track pathway encourages and educates patients to cope at home, they have the ability to contact the hospital after discharge and they receive several hours of physical therapy during the postoperative course. All baseline questionnaires were administered before the patient information and educational class, thereby including a possible effect of education in the reduction of PCS. Together with pain reduction, these factors may have been important for the reduction of pain catastrophizing in our study. Our findings suggest the need for further research regarding possible positive effects of the fast-track program.

As to be expected, the level of pain decreased over time, confirming TKA as an effective treatment for knee arthrosis [6, 27]. A higher level of preoperative pain was associated with higher postoperative pain in our study. These findings are in accordance with previous published research across different surgical fields [56]. Preoperative pain is a well-known predictor of acute and persistent postoperative pain, according to several studies [19, 57, 58].

Regarding gender, most TKAs are performed in women [59] and studies show that women are at greater risk of developing osteoarthrosis in the knee, especially after the menopause [60]. In recent years, clinical and epidemiological research has demonstrated gender differences in pain experience. Females seem to have an increased risk of persistent pain and they experience more severe clinical pain [61]. However, the results are inconclusive. Lingard et al. [62] showed that female gender had no influence on pain after TKA when adjusting for preoperative status but females had more pain at the preoperative assessment. This is in accordance with our results. When adjusting for preoperative pain, female gender was no longer statistically significant.

The study findings must be interpreted with consideration of some study limitations. First, we cannot rule out that completing the PCS questionnaire at the outpatient clinic shortly before surgery may have biased the level of catastrophizing. Second, the study cohort of 71 patients was small, but seems representative for patients scheduled for TKA as 80 % of all patients undergoing TKA during the study period were included. The sample size was comparable to other similar studies [21, 23, 36] and the response rate at each follow-up was very good. Including measures of anxiety and depression may have strengthened our study further.

## Conclusions

We found no association between preoperative catastrophizing and persistent postoperative pain in TKA patients up to one year after surgery. This may question the use of PCS as a preoperative tool to identify patients at risk for persistent pain after TKA. Further, the large reduction in pain catastrophizing scores from baseline to follow-up, both at eight weeks and one year, challenges the evidence concerning stability of pain catastrophizing over time.

### Ethics approval

As stated in the Methods section, all participants signed a written informed consent. The study was approved by the Regional Committee for Medical and Health Research Ethics in Central Norway (no. 2012/1698/REK midt).

### Consents for publications

Not applicable.

### Availability of data and materials

Request for details in the study dataset can be submitted to the corresponding author. Human subject protection requirements, appropriate data privacy as well as institutional requirements must be met.

## Abbreviations

ASA: American society of anesthesiology classification
BMI: body mass index
BPI: brief pain inventory
NRS: numerical rating scale
PCS: pain catastrophizing scale
TKA: total knee arthroplasty
